# Nicotine Dependency Levels Among Adult Electronic Cigarette Smokers in Jeddah, Saudi Arabia: An Analytical Cross-Sectional Study

**DOI:** 10.7759/cureus.61038

**Published:** 2024-05-25

**Authors:** Lama Yahya, Najlaa Mandoura, Rania Harere

**Affiliations:** 1 Preventive Medicine Postgraduate Program, Saudi Ministry of Health, Jeddah, SAU; 2 Family and Community Medicine, Saudi Ministry of Health, Jeddah, SAU

**Keywords:** e-cigarette, e-smoking, vaping, electronic cigarettes, dependency, saudi arabia, nicotine

## Abstract

Background: Electronic cigarettes (ECs) are a recent method to deliver nicotine with less harmful effects than traditional cigarettes. Studying nicotine dependence in adult EC users is a crucial area, but few measures are available to evaluate nicotine dependence induced by EC. Our study aims to estimate the levels of nicotine dependency among adult EC smokers using a modified Fagerström Test of Nicotine Dependence (e-FTND) in Jeddah, Saudi Arabia, and to identify EC-associated sociodemographic and smoking-related factors affecting nicotine dependency.

Methods: An analytical cross-sectional study was conducted on adults 18 years of age and older in Jeddah, Saudi Arabia, from December 2023 to March 2024. Data were collected from the participants using a pre-tested structured self-administered questionnaire, and nicotine dependence was assessed using the modified e-FTND. Descriptive statistics such as frequency, mean, and standard deviation were applied. Chi-square was used to assess the association between categorical variables. Ordinal regression was used to predict the nicotine dependency levels with different variables.

Results: A total of 344 participants were included in the study. The mean e-FTND score for EC users was 4.14 ± 2.45. Females had a lower likelihood of experiencing higher dependence compared to males (OR = 0.52, 95% CI: 0.32, 0.85). Using ECs for more than three years was associated with higher odds of increased dependence (OR = 3.18, 95% CI: 1.28, 7.98; p < 0.001). The use of Pod system devices lowered the odds of developing high nicotine dependence (OR = 0.32, 95% CI: 0.13, 0.75; p = 0.01) compared to Iqos device users, while mechanical Mod device users exhibited a trend towards higher dependence, although it was not statistically significant. Nicotine concentration in ECs had a significant impact on the degree of nicotine dependence. Higher concentrations were associated with increased odds of higher dependence (12-18 mg: OR = 3.26, 95% CI: 1.55, 6.91; >18 mg: OR = 4.53, 95% CI: 2.37, 8.75; p < 0.001).

Conclusion: Most exclusive EC users in the study developed a moderate nicotine dependence level. The EC device type and nicotine concentration were significant drivers of nicotine dependence. Additionally, the personal characteristics of the users, such as male gender and duration of use, were associated with a higher risk of dependence. An in-depth understanding of the magnitude of nicotine dependence among EC users will enhance the opportunity for tailored health-enhancing interventions and policies.

## Introduction

Electronic cigarettes (EC) are a recent method of delivering nicotine with fewer harmful effects than traditional cigarettes [1]. They work with battery power and produce vapor by heating liquid for inhalation [1]. The inhaled vapor may contain nicotine, flavorings, and toxins [2]. There are several primary reasons why people use them, including their potential as a smoking cessation tool and their ability to reduce cigarette consumption. This may be because the speed at which nicotine is transmitted to the brain is less rapid compared to traditional cigarettes [3, 4], helping in combating tobacco cravings and decreasing harm by substituting regular cigarettes. Finally, vaping is often viewed as a cheaper alternative to traditional cigarettes [4].

Worldwide, approximately 82 million people used ECs in 2021 [5]. A study conducted in Saudi Arabia (n=1404) revealed that 58.7% of the participants reported using ECs daily, and 35.4% stated that they used it to quit smoking [6]. Recently, the usage of ECs has become widespread among younger age groups, who are more likely to switch to smoking traditional cigarettes [7, 8]. The nicotine content in ECs is comparable to that of traditional nicotine cigarettes, which can lead to the development of nicotine dependence and the initiation of smoking cigarettes [9]. Nicotine tolerance may also develop, meaning that EC users need more nicotine to feel the same effects [10].

Furthermore, the increased trend of using both traditional cigarettes and ECs, especially among young individuals, has raised concerns about potential health hazards associated with this practice [8, 9]. Determining the level of nicotine dependence in EC users is an essential field of research. It requires in-depth analysis and examination in the context of EC users [11]. Various scores have been developed to evaluate the level of nicotine dependence in traditional tobacco cigarettes; however, very few measures are available to determine the nicotine dependence induced by ECs [11].

The modified electronic Fagerström Nicotine Dependence (e-FTND) score accurately assesses nicotine dependence and is specifically tailored to EC products [9]. It emphasizes factors associated with EC use and is one of the most prevalent scales for evaluating nicotine dependency among adult EC smokers [12].

Previous research results on nicotine dependence levels in adults who use EC have been conflicting [13, 14]. Liu et al. concluded that EC users reported lower dependence levels than traditional cigarette users [15]. However, Jankowski et al. and Jones et al. both found higher dependence levels among EC consumers [13, 14]. Additionally, previous research has mainly focused on examining the addictive effects of nicotine in traditional cigarettes and assessing the prevalence of EC use worldwide or in the Gulf region [16]. There is a lack of data regarding the nicotine dependence of ECs by validated and reliable measures in the Gulf area, especially in Saudi Arabia [17]. Our study aims to estimate the levels of nicotine dependency among adult EC smokers using a modified Fagerström score in Jeddah, Saudi Arabia, as the primary objective and to identify EC-associated sociodemographic and smoking-related factors affecting nicotine dependency.

## Materials and methods

Pilot study

A questionnaire was sent to 10% (39 subjects) of the sample participants (n = 344) via an online chat application to test the clarity of questions and identify issues with survey length. They were excluded from the primary sample and were not included in the statistical analysis.

Study design and population

Study Design

This is an analytic cross-sectional study conducted in Jeddah, Saudi Arabia, from December 2023 to March 2024.

Inclusion Criteria

The study included adults aged above 18 years who are current EC smokers, encompassing all types of what are considered ECs.

Exclusion Criteria

People using other methods of smoking more abundantly or other nicotine-delivering substances, as well as smokers who have completed a nicotine replacement therapy course, were excluded from the study.

Sampling and Sample Size

The appropriate sample size was 385 participants, calculated using Raosoft by assuming a margin of error of 5%, a confidence interval of 95%, a population size of 273,600 (according to the 2019 Kingdom of Saudi Arabia Global Adult Tobacco Survey, the prevalence of EC smokers is around 0.8%), and a response distribution of 50%.

The sampling process involved multiple stages, starting with a simple random selection of ten centers out of the 44 Ministry of Health primary healthcare centers in Jeddah city. This approach ensured that the selected sample was more representative of the general population. Each primary healthcare center was considered a cluster. The first 35 to 40 eligible participants were included from each selected primary healthcare center. Visitors and patients in each cluster were asked if they use ECs after the triage point. Those who responded positively received the questionnaire after giving their informed consent.

Data sources and collection tool

The data were collected using a pretested structured self-administered questionnaire to ensure privacy, available in both English and Arabic.

The questionnaire included sociodemographic characteristics, smoking-related behaviors, and the e-FTND [17]. It was designed to produce an ordinal measure of nicotine dependence related to cigarette smoking. The questionnaire contains six questions that evaluate the quantity of cigarette consumption, the compulsion to use, and the level of dependence [18]. In the scoring of e-FTND, yes/no items are scored from 0 to 1, and multiple-choice items are scored from 0 to 3 [11]. The higher the total e-FTND score, the more intense the patient's physical dependence on nicotine [9]. The items are summed to yield a total score of 0-10 [17]. Respondents who scored between 1 and 3 were classified as having low nicotine dependence, those who scored between 4 and 6 were classified as having moderate dependence, and those who scored between 7 and 10 were classified as having high dependence. The modified version of e-FTND used in this research, according to Piper et al. (2019), is highly correlated with validation criteria [11, 12, 18].

Statistical analysis

The data were analyzed using IBM SPSS Statistics for Windows, Version 28 (Released 2021; IBM Corp., Armonk, New York). Descriptive statistics such as frequency, mean, and standard deviation were applied. The chi-square test was used to identify any association between nicotine dependency levels and gender, nationality, marital status, educational level, occupational status, occupational domain, trying to quit smoking, device type, and flavors.

Ordinal regression was used to predict nicotine dependency levels based on gender, age, nicotine concentration, and duration of smoking, and to adjust for confounders.

Ethical considerations

Ethical approval was obtained from the Saudi Ministry of Health Institutional Review Board (IRB) with approval number A01793. Written consent was obtained at the beginning of the questionnaire from the participants. Licensed permission was obtained from the Oxford University Press journal for using the modified e-FTND score in this study.

## Results

A total of 344 subjects who currently use ECs agreed to participate and completed the questionnaire. Most were male (n=245, 71.2%) compared to females (n=99, 28.8%). The age distribution showed that the largest group was in the range of 26-35 years old (n=139, 40.4%), followed by 18-25 years old (n=97, 28.2%). The majority of participants were Saudi nationals (n=299, 86.9%).

In terms of smoking-related behaviors, more than half of the participants (n=191, 55.5%) were attempting to quit smoking. The use of ECs was quite prevalent among participants who had been smokers for more than three years (n=147, 42.7%). Disposable Pods (Pods) were the most used type of EC (n=137, 39.8%). Regarding nicotine consumption, the most frequent concentration used was between 3 and 6 mg (n=133, 38.7%). Furthermore, a significant portion of participants (n=78, 22.7%) used their ECs more than 30 times daily. Many participants (n=205, 59.6%) found it challenging to refrain from vaping in restricted areas, and a substantial number (n=241, 70.1%) continued to use their ECs even when severely ill. The description of demographics of participants who currently use ECs, as well as their behaviors, is presented in Table 1. Regarding nicotine dependence, the mean score of the e-FTND was 4.14, with a standard deviation of 2.45. Participants were further classified into the following levels: no dependence (n=24, 7%), low dependence (n=120, 34.9%), moderate dependence (n=139, 40.4%), and high dependence (n=61, 17.7%).

**Table 1:** Demographics of participants who currently use e-cigarettes, as well as their smoking-related behaviors (N=344) Data are expressed as numbers and percentages.

Sociodemographics	N (%)
Gender	
Male	245 (71.2)
Female	99 (28.8)
Age (years)	
18-25	97 (28.2)
26-35	139 (40.4)
36-45	81 (23.6)
46-55	17 (4.9)
56-65	10(2.9)
Nationality	
Saudi	299 (86.9)
Non-Saudi	45 (13.1)
Marital status	
Single	183 (53.2)
Married	152 (44.2)
Divorced	9 (2.6)
Education level	
High school or less	74 (21.5)
Bachelor	242 (70.3)
Master	20 (5.8)
PhD	8 (2.3)
Occupational status	
Unemployed	22 (6.4)
Student	54 (15.7)
Trainee	19 (5.5)
Employee	194 (56.4)
Self-employed	19 (5.5)
Manager	33 (9.6)
Retired	3 (0.9)
Occupational domain	
Unemployed/student	59 (17.2)
Entertainment	20 (5.8)
Education	29 (8.4)
Medical	40 (11.6)
Aviation	76 (22.1)
Military	13 (3.8)
Office job	80 (23.3)
Commercial	21 (6.1)
Transportation	6 (1.7)
Smoking-related characteristics	
Are you currently trying to quit smoking?	
Yes	191 (55.5)
No	153 (44.5)
For how long you have been using electronic cigarettes?	
<30 days	22(6.4)
1 month to <6 months	29(8.4)
6 months to <1 year	31(9)
1 year to < 3 years	115(33.4)
>3 years	147(42.7)
What type of electronic cigarette do you usually use?	
*Iqos* device	72 (20.9)
Disposable *Pods* device	137 (39.8)
*Pod* system device	35 (10.2)
Regulated *Mod* device	86 (25.0)
Mechanical *Mod* device	14 (4.1)
Do you use flavors?	
Yes	90 (26.2)
No	254 (73.8)
How much nicotine do you usually use?	
Less than 3 mg	84 (24.4)
3-6 mg	133 (38.7)
12-18 mg	39 (11.3)
More than 18 mg	88 (25.6)
How many times per day do you usually use your electronic cigarette?	
0-4 times/day	65 (18.9)
5-9	62 (18.0)
10-14	54 (15.7)
15-19	60 (17.4)
20-29	25 (7.3)
30+	78 (22.7)
Do you find it difficult to refrain from vaping in places where it is forbidden (e.g., in the mosque, at the library, or in the cinema)?	
Yes	205 (59.6)
No	139 (40.4)
When would you hate most to give up e-cigarette use?	
In the morning	61 (17.7)
During or after meals	69 (20.1)
During or after stressful situations	81 (23.5)
None of the above	133 (38.7)
On days that you can use your electronic cigarette freely, how soon after you wake up do you first use your electronic cigarette?	
0-5 minutes	95 (27.6)
6-15	63 (18.3)
16-30	62 (18.0)
31-60	48 (14.0)
61-120	76 (22.1)
Do you use your e-cigarette more frequently during the first two hours of the day than during the rest of the day?	
Yes	141 (41.0)
No	203 (59.0)
Do you use your e-cigarette when you are so ill (that you are in bed most of the day)?	
Yes	241 (70.1)
No	103 (29.9)
The modified Fagerstrom Test for Nicotine Dependence (mean ±SD)	4.14 ± 2.45
Nicotine dependence categories	
No dependence	24 (7.0)
Low dependence	120 (34.9)
Moderate dependence	139 (40.4)
High dependence	61 (17.7)

The chi-square analysis, presented in Table 2, explored the relationship between various factors and nicotine dependence scale categories. Males were more likely to exhibit moderate (n=118, 48.2%) and high (n=44, 18.0%) dependence, while females were more likely to exhibit low dependence (n=47, 47.5%). Age was not a significant factor (p < 0.7), with only the 18-25 age group displaying the highest proportion of high dependence (n=23, 23.7%), which, later on, in the ordinal regression, was also not statistically significant. Conversely, nationality, marital status, occupational status, and education level were not significantly associated with nicotine dependence levels (p > 0.05). On the other hand, the duration of EC use was significantly related (p < 0.001), with those using ECs for more than three years showing moderate dependence (n=73, 49.7%) and high dependence (n=36, 24.5%). 

**Table 2:** Associations between nicotine dependence levels and selected variables among adult currently E-cigarette users in Jeddah (N =344) ^1^Data are expressed as the number and percentage. ^2^The p-value was calculated using a chi-square test or Fischer exact test. *P <0.05 was significant. The scores of the e-FTND were categorized as follows: scores between 1 and 3 indicated a low nicotine dependence level, scores between 4 and 6 indicated a moderate dependence level, and scores between 7 and 10 indicated a high dependence level.

	No dependence, N = 24^1^	Low dependence, N = 120^1^	Moderate dependence, N = 139^1^	High dependence, N = 61^1^	p-value^2^
Gender					<0.001*
Male	10 (4.1)	73 (29.8)	118 (48.2)	44 (18.0)	
Female	14 (14.1)	47 (47.5)	21 (21.2)	17 (17.2)	
Age (years)					0.7
18-45	23(7.3)	111(35.0)	126(39.7)	57 (18.0)	
More than 45 years	1(3.7)	9(33.3)	13(48.2)	4(14.8)	
Nationality					0.3
Non-Saudi	6 (13.3)	13 (28.9)	19 (42.2)	7 (15.6)	
Saudi	18 (6.0)	107 (35.8)	120 (40.1)	54 (18.1)	
Marital Status					0.2
Single	13 (7.1)	65 (35.5)	72 (39.3)	33 (18.0)	
Married	11 (7.2)	55 (36.2)	62 (40.8)	24 (15.8)	
Divorced	0 (0.0)	0 (0.0)	5 (55.6)	4 (44.4)	
Education Level					0.3
High school or less	4 (5.4)	21 (28.4)	33 (44.6)	16 (21.6)	
Bachelor	17 (7.0)	87 (36.0)	99 (40.9)	39 (16.1)	
Master	1 (5.0)	8 (40.0)	6 (30.0)	5 (25.0)	
PhD	2 (25.0)	4 (50.0)	1 (12.5)	1 (12.5)	
Occupational Status					0.9
Unemployed	3 (13.6)	7 (31.8)	8 (36.4)	4 (18.2)	
Student	4 (7.4)	22 (40.7)	19 (35.2)	9 (16.7)	
Trainee	1 (5.3)	7 (36.8)	8 (42.1)	3 (15.8)	
Employee	14 (7.2)	62 (32.0)	81 (41.8)	37 (19.1)	
Self-employed	0 (0.0)	8 (42.1)	8 (42.1)	3 (15.8)	
Manager	2 (6.1)	14 (42.4)	13 (39.4)	4 (12.1)	
Retired	0 (0.0)	0 (0.0)	2 (66.7)	1 (33.3)	
Currently trying to quit smoking.					0.5
Yes	10 (5.2)	69 (36.1)	76 (39.8)	36 (18.8)	
No	14 (9.2)	51 (33.3)	63 (41.2)	25 (16.3)	
Duration of using EC					<0.001*
Less than 30 days	4 (18.2)	11 (50.0)	4 (18.2)	3 (13.6)	
1 to 6 months	3 (10.3)	13 (44.8)	10 (34.5)	3 (10.3)	
6 months to 1 year	1 (3.2)	11 (35.5)	16 (51.6)	3 (9.7)	
1 year to 3 years	14 (12.2)	49 (42.6)	36 (31.3)	16 (13.9)	
More than 3 years	2 (1.4)	36 (24.5)	73 (49.7)	36 (24.5)	
Type of EC					<0.001*
Iqos device	3 (4.2)	25 (34.7)	34 (47.2)	10 (13.9)	
Disposable Pods device	9 (6.6)	52 (38.0)	51 (37.2)	25 (18.2)	
Pod system device	8 (22.9)	16 (45.7)	9 (25.7)	2 (5.7)	
Regulated Mods device	3 (3.5)	25 (29.1)	41 (47.7)	17 (19.8)	
Mechanical Mods device	1 (7.1)	2 (14.3)	4 (28.6)	7 (50.0)	
Do you use flavors?					0.6
Yes	15 (5.9)	88 (34.6)	104 (40.9)	47 (18.5)	
No	9 (10.0)	32 (35.6)	35 (38.9)	14 (15.6)	
How much nicotine do you usually use?					<0.001*
Less than 3 mg	12 (14.3)	41 (48.8)	26 (31.0)	5 (6.0)	
3-6 mg	11 (8.3)	51 (38.3)	51 (38.3)	20 (15.0)	
12-18 mg	1 (2.6)	9 (23.1)	21 (53.8)	8 (20.5)	
More than 18 mg	0 (0.0)	19 (21.6)	41 (46.6)	28 (31.8)	

Regarding the type of EC, half the users of mechanical Mod devices (n=7, 50%) demonstrated a high nicotine dependence level (p < 0.001). Additionally, there was a strong association between nicotine concentration and nicotine dependence (p < 0.001), with higher concentrations being linked to greater dependence, especially among individuals using more than 18 mg (n=28, 31.8%).

The ordinal regression analysis investigated the main factors related to nicotine dependence among participants who use EC; the results are presented in Table 3. Associations between dependence levels and gender, duration of EC use, type of device, and nicotine concentration were shown to be statistically significant. The findings indicated that gender has a significant impact on nicotine dependence levels (p < 0.001). Specifically, females had a lower likelihood of experiencing higher dependence than males (OR = 0.52, 95% CI: 0.32, 0.85). The age group (18-45 years) and attempts to quit smoking did not significantly influence the levels of dependence. 

The duration of EC use was a significant predictor, with those using for more than three years having higher odds of increased dependence (OR = 3.18, 95% CI: 1.28, 7.98; p < 0.001). The type of EC also had an impact on nicotine dependence; the use of Pod system devices lowered the odds of developing high nicotine dependence (OR = 0.32, 95% CI: 0.13, 0.75; p = 0.01) compared to Iqos device users, while mechanical Mod device users exhibited a trend towards higher dependence, although it was not statistically significant. Nicotine concentration in ECs had a significant impact on the degree of nicotine dependence. Higher concentrations were associated with increased odds of higher dependence (12-18 mg: OR = 3.26, 95% CI: 1.55, 6.91; >18 mg: OR = 4.53, 95% CI: 2.37, 8.75; p < 0.001).

**Table 3:** Demographics and EC-related behaviors associated with e-FTND score by ordinal regression analysis. * P < 0.05 was considered significant. OR: odds ratio, CI: confidence interval, EC: e-cigarette, e-FTND: Fagerström Test of Nicotine Dependence.

Characteristics	OR	95% CI	P value
Gender			<0.001*
Male	–	–	
Female	0.52	0.32, 0.85	
Age			0.83
18-45	–	–	
>45	1.08	0.50, 2.31	
Currently trying to quit smoking.			0.30
No	–	–	
Yes	1.10	0.72, 1.67	
What is the duration of using electronic cigarettes?			
<30 days	–	–	
1 month to <6 months	1.34	0.45, 4.00	0.60
6 months to <1 year	2.53	0.86, 7.47	0.09
1 year to <3 years	1.24	0.49, 3.13	0.14
>3 years	3.18	1.28, 7.98	<0.001*
What type of electronic cigarette do you usually use?			
Iqos device	–	–	
Disposable Pods devices	0.87	0.47, 1.59	0.65
Pod system devices	0.32	0.13, 0.75	0.01*
Regulated Mods devices	0.66	0.33, 1.33	0.25
Mechanical Mods devices	3.10	0.91, 11.1	0.07
Do you use flavors?			
No	–	–	
Yes	1.28	0.74, 2.20	0.25
How much nicotine do you usually use?			
<3 mg	–	–	
3-6 mg	1.54	0.90, 2.68	0.37
12-18 mg	3.26	1.55, 6.91	<0.001*
More than 18 mg	4.53	2.37, 8.75	<0.001*

## Discussion

The current study aimed to assess the levels of nicotine dependency and the sociodemographic and smoking-related predictors of dependence among exclusive EC adult users. Most participants were categorized as moderately dependent. Gender, length of EC usage, EC device types, and nicotine concentration were significantly associated with nicotine dependence levels.

In the present study, the mean FTND score for EC users was 4.14 ± 2.45. González Roz et al. reported a similar mean score among Spanish EC users (4.38 ± 1.93), while traditional cigarette smokers showed a higher dependence score (mean FTND score = 5.57 ± 1.48) [19]. Nicotine dependence was high in 61 (17.7%) participants, moderate in 139 (40.4%), and low in 120 (34.9%) of the present study responders. The study's dependence levels among EC users are comparable to earlier research in the United States and Colombia, even though the studies included dual users [19, 20].

The fact that over half of the responders attempted to quit smoking aligns with ECs' widespread marketing as a smoking cessation aid; however, the evidence of their use in tobacco cessation is questionable [21, 22]. According to the Cochrane Review in 2024, evidence for EC efficacy in smoking cessation was highly certain compared to nicotine replacement therapy but less certain when contrasted with usual or no care [23]. Although there was no evidence of serious adverse events among nicotine EC users, the study was limited by the short follow-up duration and the small number of randomized controlled studies [23].

Specific EC device features were significantly linked to nicotine dependence in our analysis. Similar to earlier research among 3,609 EC users, the current study indicated a positive association between the e-liquid's nicotine concentration (≥12 mg) and the dependence level [12]. Dependence was linked to higher e-liquid nicotine concentrations used by naive EC smokers [24]. By contrast, a pilot study demonstrated no significant relationship between nicotine concentration and EC dependence scale among users of disposable and pod-based EC devices [25]. These contradictory findings may be clarified by the variability in devices’ nicotine flux, as it was significantly associated with dependence scores among pod-based EC users [26].

Compared to Iqos device users, users of pod-based devices reported lower dependence levels after controlling for other variables in our study. On the other hand, higher nicotine dependence was associated with the use of pod devices in samples of adolescents and adults [16, 27, 28]. A recent study reported no significant impact of EC device type on the dependence levels among American youth and young adult consumers [29]. The variety of EC devices did not influence the dependency scores among adult nonsmoking EC users [24]. Although the mechanisms for varying dependence risks across devices need further research, the variation in sample size, population, and tobacco cigarette usage patterns may be attributed to the heterogeneity across the studies.

Males in our sample showed a significantly higher risk of nicotine dependence, which is in line with earlier research among Malaysian EC consumers [8]. A three-year cohort study revealed that gender was not linked to higher odds of starting electronic nicotine products, although males were more prone to frequent use upon initiation [30]. Frequent EC use was associated with a higher nicotine dependence score [8]. Future studies are needed to investigate the gender-associated drivers of nicotine dependence among EC users.

Our investigation identified the usage of ECs for more than three years as a risk factor for nicotine dependence. Similarly, a study including participants aged 16 to 25 years indicated that EC consumers showed symptoms of dependence after two to five years of usage [31]. Additionally, Foulds et al. observed the association between increased nicotine dependence and the length of EC use [12].

To our knowledge, this is the first research on EC dependence and associated factors in Saudi Arabia. Another strength of the study is the exclusive inclusion of EC users, which excludes confounders related to concurrent nicotine dependence. Furthermore, the study was multicentered in Saudi Arabia's second-largest city, Jeddah. Certain limitations need to be considered when interpreting the study. First, the cross-sectional design limits the study's ability to confirm causality. Second, the study's limitation to one city may restrict its generalizability. Third, the use of self-reported data increases the risk of recall bias. Fourth, the study omitted the device power data, which modulates nicotine delivery in addition to EC device features such as nicotine concentration [32].

## Conclusions

Most exclusive EC users in the study developed a moderate dependence level. The EC device type and nicotine concentration were significant drivers of nicotine dependence. Additionally, the personal characteristics of the users, such as male gender and duration of use, were associated with a higher risk of dependence. An in-depth understanding of the magnitude of nicotine dependence among EC users will enhance the opportunity for tailored health-enhancing interventions and policies. Future longitudinal studies are needed to explore the burden of dependence among EC users, including biological biomarkers as measures of nicotine dependence.
